# Evaluation of grouped capsule network for intracranial hemorrhage segmentation in CT scans

**DOI:** 10.1038/s41598-023-30581-4

**Published:** 2023-03-01

**Authors:** Lingying Wang, Menglin Tang, Xiuying Hu

**Affiliations:** 1 West China School of Nursing, Sichuan University/ West China Hospital Critical Care Medicine Department, Sichuan University, Chengdu, 610041 China; 2 Innovation Center of Nursing Research, Nursing Key Laboratory of Sichuan Province, West China Hospital, Sichuan University, Chengdu, 610041 China

**Keywords:** Machine learning, Image processing, Cerebrovascular disorders

## Abstract

Intracranial hemorrhage is a cerebral vascular disease with high mortality. Automotive diagnosing and segmentation of intracranial hemorrhage in Computed Tomography (CT) could assist the neurosurgeon in making treatment plans, which improves the survival rate. In this paper, we design a grouped capsule network named GroupCapsNet to segment the hemorrhage region from a Non-contract CT scan. In grouped capsule network, we constrain the prediction capsules for output capsules produced from different groups of input capsules with various types in each layer. This method can reduce the number of intermediate prediction capsules and accelerate the capsule network. In addition, we modify the squashing function to further accelerate the forward procedure without sacrificing its performance. We evaluate our proposed method with a collected dataset containing 210 intracranial hemorrhage CT scan slices. In experiments, our proposed method achieves competitive results in intracranial hemorrhage area segmentation compared to the existing methods.

## Introduction

Intracranial hemorrhage, which causes bleeding within brain tissue, is a life-threatening cerebral disease with high incidence and mortality rates^1,2^. Non-contrast Computed Tomography (CT) scan is the first imaging modality to diagnose intracranial hemorrhage(ICH), due to its rapidity, convenience, and sensitivity to blood. The location and volume of hemorrhage derived from CT are crucial for neurosurgeons to make operation plans. The volume of ICH can be a predictor of mortality^3^ and secondary hemorrhage expansion (HE)^1,4,5^. Automatic intracranial hemorrhage segmentation on CT^4,6,7^ is a fundamental task that assists radiologists in the quantification of hemorrhage volume and shapes analysis.

Recent developments show the deep convolutional neural network (CNN) is powerful in medical image analysis^8^. Some existing methods pay attention to the following related problem: ICH or normal detection^9–11^, ICH sub-types detection^12–14^ and hemorrhage region segmentation^14–17^. These CNN models have significantly improved performance by providing frameworks for end-to-end representation learning and a large amount of data training. Then, a validation or test data set is used to evaluate their generalization performance. These methods benefit from the powerful feature extraction ability of convolution neural networks and achieve promising performance.

However, existing CNN-based methods tend to suffer performance degradation due to the lack of spatial relationships or a large amount of data. Beyond CNN, the capsule network extends using vector^18–20^ or matrix^21–23^ as a representative unit instead of scalar in CNN. Vector preserves equivariance through the whole capsule network, where its length and orientation represent the probability and property of entity. The evidence that the capsule network has a more powerful representative ability than CNN is shown in the initial experiment^18,24^. While sharing the weights and improving the dynamic routing algorithm, these methods remain to have high computational overhead. How to reduce the amount of computation while maintaining the segmentation performance of the capsule network is still an open problem.

In this paper, we propose an effective approach, referred to as GroupCapsNet for intracranial hemorrhage segmentation. Firstly, inspired by group convolution^25^, which is widely used to reduce convolution calculation, we introduce the group concept to accelerate the capsule network and reduce the memory consumption of immediate vote capsules. During the capsule forwarding procedure, the output capsule only receives a group of input capsules which is a proportion of the whole input. Then, we further propose to modify the squashing function to have a faster computing speed without diminishing the performance.

### Contributions

In this paper, our contributions can be summarized as follows.

1. We propose the group capsule to accelerate the capsule network and reduce the memory consumption of immediate vote capsules. The modified squashing function also suggests to replace the original one.
2. Our GroupCapsNet achieves comparable results with the existing methods in intracerebral hemorrhage segmentation tasks. Comprehensive experiments are conducted to show the superior performance of the group capsule network compared to the non-group counterpart and the convolutional neural network.

## Related work

In this section, we will briefly highlight some recent related work including ICH region segmentation, capsule network, and group concept used in CNN.

### ICH region segmentation

Either segmentation or dense prediction on the voxel level of ICH is required to calculate the volume of ICH. Grad-CAM^26^ used by Ye et al.^12^ can be employed to produce a highlighted map for ICH, but this map is too coarse and just be adopted as guidance for the interpretation of neural networks by radiologists. A more accurate ICH segmentation map can be obtained by training a full convolution network (FCN) in a supervised learning manner. Chilamkurthy et al.^14^ used U-Net based approach to segment ICH, intraparenchymal (IPH), intraventricular (IVH), subdural (SDH), extradural (EDH) and subarachnoid (SAH) hemorrhages Manvel et al.^17^ explored the variant U-Net method with test time augmentation and ensembling strategies to segment ICH in three projections (i.e., axial, coronal, and sagittal). Islam et al.^16^ proposed ICHNet facilitating hypercolumn features from dilated convolutional layer and incorporating 3D conditional random field to smoothen the segmentation prediction. Ironside et al.^15^ evaluated a deeper variant U-Net in ICH volumetric analysis. These methods are based on CNN (especially U-Net^8^), although there are some inherent shortcomings in CNN^18^.

### Capsule network

In the capsule, the feature is stored as vector^18^ or matrix^21^ to preserve its equivariance. The length and orientation of the vector represent the probability and property of the entity presented in the input. In forwarding capsules to the next layer, each input capsule produces a vote capsule to each capsule in the next layer by matrix transformation. This procedure requires additional computational cost and large memory consumption, resulting in the inability to apply a larger and deeper capsule network. To tackle this drawback of the capsule, trainable weight matrices within the local spatial kernel of the same type of capsule are reasonably shared^18,27^. In addition, capsule-pooling layer^28^ was proposed in generalizing capsules to higher dimensional inputs. Are the variants of dynamic routing or expectation-maximum (EM) routing based on weighted kernel density estimation^29^ was came up with a faster and more effective procedure. These methods enable the capsule network to maintain performance while reducing computational costs. The capsule has also been extended and studied to segmentation tasks by some researchers^27,30^. However, because of the heavy memory consumption of immediate vote capsules and the expansive computation of the dynamic routing algorithm, the capsule network is hard to become as deep and large as CNN is. To conquer the issue and apply it to large data set and explore the potential, many researchers focus on accelerating and simplifying the capsule network by sharing the weights^18,27,28^, introducing special capsule layer^28,31^, improving the dynamic routing algorithm^32,33^ etc. Particularly, SegCaps^27,34,35^ extend vector capsule to object segmentation including intracranial hemorrhage segmentation task. Despite the accuracy, capsules are less efficient to large computing overhead. How to maintain the accuracy while improving the computational efficiency of the capsule network is still an open problem.

## Methods

In this section, we will introduce our capsule network (named GroupCapsNet) used in ICH segmentation. First, we will briefly revisit the basic capsule concept. Second, the grouped capsule on which GroupCapsNet is built will be illustrated and discussed. Finally, the whole GroupCapsNet will be described including its special layer and loss function.

### Ethical approval

The study was approved by the Ethics Committee of West China Hospital, Sichuan University in 2022. The study was performed in accordance with relevant guidelines and regulations in compliance with the Declaration of Helsinki. All images were from HIS cases of the system. Informed consent was obtained from all participants and the data was treated anonymously.

### Recap capsule

The capsule is a novel form of feature representation, which makes use of vector^18^ or matrix^21^ as its unit instead of scalar in a neural network. For clarity, we use vector capsule by default. As to forwarding propagation, it has a similar manner in both capsule and neural networks. The next layer of the capsule will be produced in the next three steps, voting, clustering, and nonlinear mapping.

Let $v^L_t \in {\textbf{R}}^{n \times 1}$ denote the type *t* capsule in layer *L*. In voting step, each capsule $v^L_t$ produces its vote capsule $u_{t'|t}^{L+1} \in {\textbf{R}}^{n' \times 1}$ for type $t'$ capsule in layer $L+1$ by matrix transformation as follows:

1 $$\begin{aligned} u_{t'|t}^{L+1} = W_{t'|t}^L \cdot v_t^L \end{aligned}$$

where $W_{t'|t}^L \in {\textbf{R}}^{n' \times n}$ is a trainable weight matrix.

In clustering step, all vote capsules $u_{t'|t}^{L+1}$ are aggregatd by weighted summation to produce the dominant capsule ${\hat{v}}^{L+1}_{t'}$ as follows:

2 $$\begin{aligned} {\hat{v}}^{L+1}_{t'} = \sum _{t} c_{t'|t}^{L+1} u_{t'|t}^{L+1} \end{aligned}$$

where $c_{t'|t}^{L+1} \in {\textbf{R}}$ is the weight and can be derived from routing algorithm^18^.

In nonlinear mapping step, dominant capsule ${\hat{v}}^{L+1}_{t'}$ is mapping by a squashing function to obtain the final output capsule $v^{L+1}_{t'}$. The squashing function can be formulated as follows:

3 $$\begin{aligned} v^{L+1}_{t'} = \frac{\left\| {\hat{v}}^{L+1}_{t'}\right\| ^2}{1+\left\| {\hat{v}}^{L+1}_{t'}\right\| ^2} \cdot \frac{{\hat{v}}^{L+1}_{t'}}{\left\| {\hat{v}}^{L+1}_{t'}\right\| } \end{aligned}$$

#### Convolutional capsule

For inputs like images, the capsule can take advantage of the local receptive field and weight sharing like the convolutional neural network (CNN). The convolutional capsule is obtained from different types of capsules within a spatial local kernel in the previous layer. Additionally, a trainable weight matrix of the same type of capsules is reasonably shared at a different spatial locations to further reduce the computational cost^18,27,28^.

### Grouped capsule

Different types of capsules are distinguishing features of different entities or parts. To produce a capsule, it does not need all types of capsules in the previous layer as input. The distinguishing feature requires relevant types of capsules rather than as many as possible types of capsules. Therefore, it is reasonable to divide different types of the capsule into groups. In addition, grouped computation in a convolutional neural network has been more widely studied recently and empirically demonstrated its potential in improving accuracy and reducing computational cost^36^.

**Figure 1:**
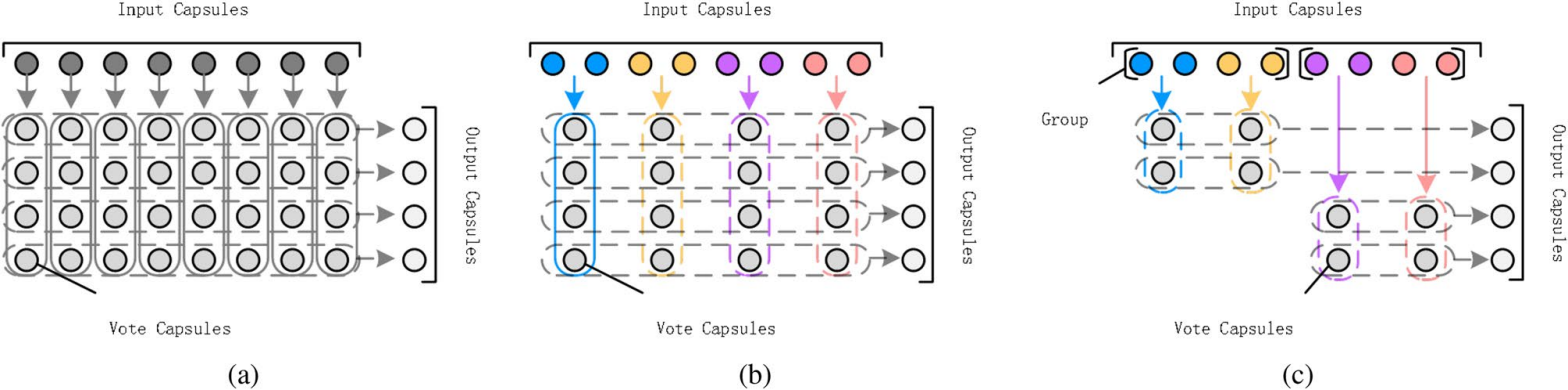
Demonstration of capsules forwarding between layers. (**a**) Each input capsule produces a vote capsule for output capsule. (**b**) Trainable weight matrics are shared within local spatial kernel of the same type capsules. Thus, the capsules in the same type only produce one capsule for each output capsule. (**c**) Grouped capsule.

Based on the assumption that the relevant types of the capsule can be automatically gathered into one group by training procedure, we equally divide all types of the capsule into non-overlapping groups. Then, each group of capsule independently produce the capsule in the next layer (Fig. 1).

Formally, in grouped capsule, all types *t* of capsules in one layer are divided into *g* (i.e. 2, 4 or 8) groups denoted as $G = \{ G_1, G_2, \dots , G_g \}$, where $G_i \subset \{t\}^n$ and $| G_i | = | G_j |$, $G_i \cap G_j = \emptyset$ for every $i\ne j$. For the next layer, capsules with type $t'$ are also divided into $G' = \{ G'_1, G'_2, \dots , G'_g \}$ satisfying the same constraint as *G*, where $G'_i \subset \{t'\}^n$. Capsule with type $t' \in G'_i$ is only related to type $t \in G_i$ capsule in the previous layer, therefore Eq. (2) becomes as follows:

4 $$\begin{aligned} {\hat{v}}^{L+1}_{t'} = \sum _{t\in G_i} c_{t'|t}^{L+1} u_{t'|t}^{L+1} \end{aligned}$$

where $t' \in G'_i$. On the other hand, capsule with type *t* only produces the vote capsules for types in $G'_i$ which is a portion of all types. This ends up with a significant reduction of both the trainable weight matrix and immediate vote capsules. Theoretically, when *g* increases, the computational cost reduces and is roughly 1/*g* original without grouped capsule. This forces the grouped capsule to learn the compact representative features.

The grouped capsule can easily generalize to the convolutional version in the case of image-like inputs, where the capsule only has the connection to the previous layer capsules within a specific kernel. Furthermore, the grouped capsule can be seamlessly added to the existing capsule network.

### Network architecture

Our whole network architecture named GroupCapsNet is based on a symmetrical U-shape design following U-Net^8^. GroupCapsNet contains the encoder and the decoder parts. The encoder part extracts high-level semantic features, while the decoder part restores these features in high spatial resolution.

In the encoder part, two normal convolutional layers with 16 channels, $3 \times 3$ kernel and stride 1 are firstly applied. We regard 16-channels convolutional output as 2-types, 8-dimensions capsules. Non-linear mapping is employed to the output of convolutional layers to form the initial capsules before forwarding to the next step. Then, the initial capsules are processed through four stages. The design for these stages follows three rules: capsules in the same stage have the same number of types and dimensions; capsules in the next stage double the number of types and dimensions, and halve the spatial resolution by $2 \times 2$ stride;the number of grouped capsule layer doubles in the next stage.

In the decoder part, there also exist four stages. At each stage, the deconvolutional capsule is firstly applied to double spatial resolution from the output of the previous stage or the final stage in the encoder part. All types of the capsule from the output of the deconvolutional capsule and the corresponding stage in the encoder part are collected for subsequent processing. These stages are also satisfied the similar rules as mentioned in the encoder part: capsules in the same stage have the same number of types and dimensions; the number of grouped capsule layer halves in the next stage.

The segmentation header layer is added up to the output of the decoder. In the segmentation header, one capsule layer with $1 \times 1$ kernel and $1 \times 1$ stride is employed to produce the final 1-type, 8-dimension segmentations capsules. The hemorrhage region and the background are determined by the length of segmentation capsules after thresholding.

#### Non-linear mapping

Non-linear mapping is designed to enhance the non-linear representation ability of the network. The squashing function (Eq. 3) normalizes the length of the capsule. The right part of the squashing makes the capsule become a unit vector, while the left part takes non-linear mapping to its length. In our GroupCapsNet, we propose to use the modified squashing function as follows:

5 $$\begin{aligned} v^{L+1}_{t'} = \frac{\left\| {\hat{v}}^{L+1}_{t'}\right\| }{1+\left\| {\hat{v}}^{L+1}_{t'}\right\| } \cdot \frac{{\hat{v}}^{L+1}_{t'}}{\left\| {\hat{v}}^{L+1}_{t'}\right\| } = \frac{{\hat{v}}^{L+1}_{t'}}{1+\left\| {\hat{v}}^{L+1}_{t'}\right\| } \end{aligned}$$

which is slightly different from the left part of the squashing function.

## Results

In this section, we will conduct comprehensive experiment to evaluate our GroupCapsNet in ICH segmentation. First, we will describe the experimental setting including data set collection, preprocessing and metric involved in our experiment. Second, ablation study of GroupCapsNet is conducted to demonstrate the effectivness of group number and modified squashing function. Finally, we discuss the comparison of GroupCapsNet and the existing methods in ICH Segmentation.

### Experimental setting

#### Implementation details

We implement our proposed method with PyTorch on NVIDIA Tesla V100 GPUs. We perform random flip, rotation as data augmentation and gaussian additive noise with ${\mathcal {N}}(0,0.01)$ as data perturbation. Our method is trained by Adam optimizer^37^ with initial learning rate $5e-4$ for 100 epochs. The learning rate is reduced by factor 0.97 for each epoch. The batch size is 32 where a half is labeled images and the rest is unlabeled images. We set $\alpha =0.99$ in exponential moving average(EMA).

#### Dataset

The dataset used in this experiment is a non-public dataset which is came from our cooperative hospital, which consists a total of 210 Non-Contrast CT scans of patients’ brains from cooperative hospital during January 2017 to December 2019 named ICH210. Each slice of CT scans has been traced into hemorrhage and brain tissues by a master degree student who majors in neurosurgery and all the tracing result have been checked by an emergency medicine specialist working in a hospital. The size of Non-Contrast CT scans is ${\mathcal {R}}^{512 \times 512 \times (10 - 60)}$. We employ 5-fold cross validation technology to evaluate our proposed method.

#### Preprocessing

Because the brain tissues and hemorrhage that we concerned have a CT value smaller than 90 Hounsfield Unit, we clip all scans CT value into the range of 0–90 and then normalize them into 0–1. We split the CT scans into slices in z-axial. Slice images have been resized into ${\mathcal {R}}^{256 \times 256}$. For data augmentation, we flip and rotate the slice randomly.

#### Evaluation metric

To quantitatively evaluate the methods in ICH segmentation, we calculate four types metrics: Dice Coefficient (Dice), Intersection over Union (IOU), Sensitivity and Specificity^16^. They can be formulated as follows:

6 $$\begin{aligned} Dice= & {} \frac{2 \cdot TP}{2 \cdot TP+FN+FP} \end{aligned}$$

7 $$\begin{aligned} IOU= & {} \frac{TP}{TP+FN+FP} \end{aligned}$$

8 $$\begin{aligned} Sensitivity= & {} \frac{TP}{TP + FN} \end{aligned}$$

9 $$\begin{aligned} Specificity= & {} \frac{TN}{TN + FP} \end{aligned}$$

where *TP*, *FP*, *TN* and *FN* are the number of true-positive, false-positive, true-negative and false-negative respectively. Due to common data imbalance problems invovled in ICH segmentation, the performance of segmentation mainly depends on Dice Coefficient.

### Ablation study

#### Modified squashing vs. squashing

**Figure 2:**
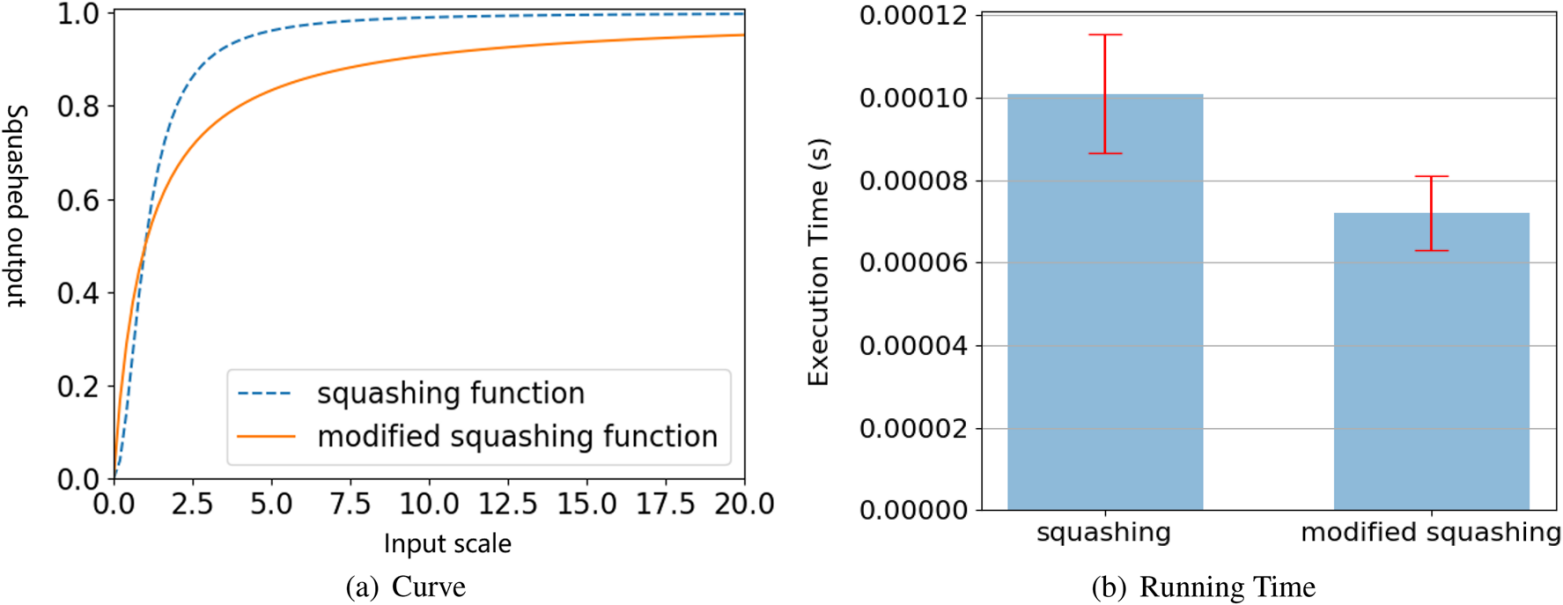
Different types of non-linear mapping function curve.

The only difference between modified squashing and squashing function is how to take non-linear mapping on capsule length. By rewriting Eq. (3) into Eq. (5), these non-linear mapping function have the similar properties as their curves shown in Fig. 2a. However, modified squashing has a greater advantage over squashing in term of forward running time. We evaluate them by a 16-dimension capsule and their forward execution times are shown in Fig. 2b. The result is obtained by averaging over 1000 times experiments on PyTorch platform. The forward time of the modified squashing is about 30% lower than that of the squashing (from 0.0001 to 0.00007 s). Because the modified squashing reduces the number of floating point operations, in our experiment, the backward running time of the modified squashing is also reduced.

We conduct the experiment to compare the impact of different non-linear mapping function on performance. We train two types of the GroupCapsNet about 250 epoches in ICH210 dataset. As shown in Table 1, comparing to squashing, modified squashing doesn’t reduce but slightly improve the performance of the GroupCapsNet.

**Table 1:** Performance comparison of different non-linear mapping function.

	Non-linear function	Dice (%)	IOU (%)
GroupCapsNet	Squashing	87.02	76.15
GroupCapsNet	Modified squashing	87.26	76.34

#### Grouped vs. non-grouped

We investigate the impact of grouped capsule and non-grouped capsule in terms of computational speed and performance. The number of groups is a crucial factor in our GroupCapsNet. When the group number *g* increases, the number of immediate vote capsules significantly reduces by 1/*g*. This is beneficial in computational speed of both forward and backward procedures between capsules. We note that non-grouped capsule network can be seen as a special case of grouped capsule network (i.e. $g=1$).

**Figure 3:**
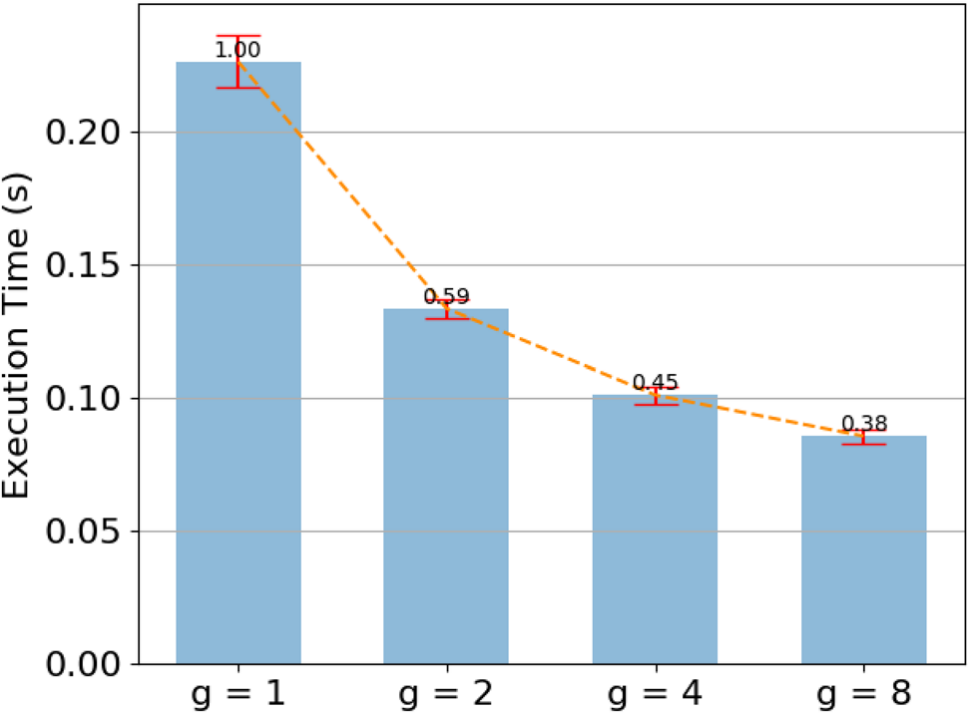
Execution time of different group number.

To empirically show the impact of the number of group in computational speed, we study four types of grouped capsules with different group number (i.e. $g=1$, $g=2$, $g=4$ and $g=8$). For different types of grouped capsules, the dimension of input and output capsules are 16 and 32 respectively, and the dimension of other capsules are all 8. The spatial resolution of different grouped capsule is $64 \times 64$ and the iteration number of routing algorithm is 3. The experiment result is shown in Fig. 3. The forward execution times of $g=2$, $g=4$ and $g=8$ grouped capsule are 59%, 45% and 38% of non-grouped capsule. This shows that grouped capsule can significantly reduce the computational cost. We note that the number of execute time reduces is not the theoretical value (i.e. 1/*g*). The reason may be the inefficient operation of concatenating all capsule groups to form the output capsule. When *g* increases, the proportion of operation time increases.

To investigate the impact of the group number on the performance, we design four types of GroupCapsNets, which have different group numbers in grouped capsule layer. When the numbers of initial capsule types and dimensions are determined, the following architecture of GroupCapsNet can be automatically configurated due to its highly modular design. We denote four types of GroupCapsNets as GroupCapsNet-G1, GroupCapsNet-G2, GroupCapsNet-G4 and GroupCapsNet-G8, which have the group number 1, 2, 4 and 8 respectively. And we set the numbers of initial capsule types and dimensions in GroupCapsNet-G$\#g$ ($\#g \in \{1, 2, 4, 8\}$) as $\#g$ and $16/\#g$ respectively. We train these types of GroupCapsNets about 250 epoches in ICH210 dataset. The experiment results are shown in Table 2. GroupCapsNet-G2 achieves the best performance and outperforms 2% than non-grouped GroupCapsNet in term of dice coefficient. The results also show that the performance decreases when $g=4, 8$. On the one hand, we think that how many groups capsules can be divided depends on specific dataset. If we force to split the relevant types capsules into independent groups, the effect of GroupCapsNet will decline. On the other hand, when $g=4,8$, the couterpart of GroupCapsNet have fewer weights to capture patterns and the capsules with fewer dimension may do not have sufficient representation capacity.

**Table 2:** Performance comparison of GroupCapsNets with different group numbers.

	$\#g$	$\#weight$	DSC (%)
GroupCapsNet-G1	1	4.86 M	85.04
GroupCapsNet-G2	2	2.77 M	87.26
GroupCapsNet-G4	4	1.75 M	85.72
GroupCapsNet-G8	8	1.34 M	80.98

### Compare with existing methods

To demonstrate the effectiveness of GroupCapsNet, we compare it with existing methods of ICH segmentation. DPN92-Unet and ICHNet are recently proposed to focus on ICH segmentation and achieve promising results. These models are based on deep convolutional nerual network, unfortunately, their datasets and code are not public.

U-Net is a mature medical image segmentation method, which is simple but effective, and it is still state-of-the-art in some applications recently. We evaluate a smaller version of U-Net named U-Net-2M on ICH210, which has 1/4 trainable weight parameters in each layer comparing to the original U-Net. U-Net-2M is designed to have similar parameters as our GroupCapsNet. The SegCaps is also evaluated on ICH210, because it is similar to our non-group counterpart of GroupCapsNet. U-Net-2M, SegCaps and GroupCapsNet are trained using BCE loss. The optimizer is Adam with 0.001 learning rate. The learning rate is decayed by a factor of 0.9 for 60 epoches. All the methods are trained at most 250 epoches. Table 3 shows the result from 5-folds cross validation of different methods. We also conduct an ensembles of 3 GroupCapsNets.

**Table 3:** Performance comparison between GroupCapsNet and existing methods in intracranial hemorrhage segmentation, DPN92-Unet ensembles and GroupCapsNet ensembles methods means that training 3 models and averaging their prediction results.

	$\#$CT Scans	Cross Validation	Dice	Sensitivity	Specificity
DPN92-Unet	210	Y	0.6830	–	–
DPN92-Unet ensembles	210	N	0.7300	–	–
ICHNet	210	Y	0.8760	0.9151	0.9984
U-Net 2M	210	Y	0.8569	0.9020	0.9991
SegCaps	210	Y	0.8582	0.8981	0.9985
GroupCapsNet (ours)	210	Y	0.8726	0.9133	0.9989
GroupCapsNet (ours)	413	Y	0.8894	0.9208	0.9983
GroupCapsNet ensembles	210	N	0.8901	0.9214	0.9981

Our GroupCapsNet outperforms U-Net 2M by about 2% in term of Dice. In the case of the similar number of parameters, GroupCapsNet shows its potential on feature representation. The SegCaps is one kind of non-group capsule network. The experiment shows the effectiveness of grouped capsule. In addition, our GroupCapsNet shows the comparable performance as comparing to ICHNet in ICH segmentation. After ensembling (training 3 models and averaging their prediction results), our GroupCapsNet achieve the best dice of 0.8901 in ICH segmentation.

**Figure 4:**
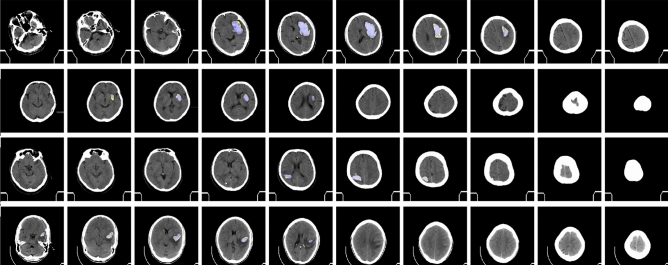
Samples of ICH segmentation prediction from GroupCapsNet and label hemorrhage region. (Best viewed in color and with zoom in).

The quantitative output for ICH segmentation is shown in Fig. 4.

## Conclusion

In this paper, we aim to reduce the burden computational cost and memory consuming of capsule network and employ it to ICH segmentation task. To solve the shortcoming of capsules, we design a capsule network named GroupCapsNet comprising the grouped capsule layer. A comprehensive experiment is conducted to show that the effectivness of grouped capsule and modified squashing function. In ICH segmentation, our GroupCapsNet achieve the comparable performence as comparing to the existing approaches.

## Data Availability

The datasets analysed during the current study are not publicly available due to protection of personal information but are available from the corresponding author on reasonable request.
